# Antivirotics based on defective interfering particles: emerging concepts and challenges

**DOI:** 10.3389/fcimb.2025.1436026

**Published:** 2025-02-24

**Authors:** S. V. Maryanchik, S. E. Borovikova, A. O. Ivanova, V. V. Trofimov, O. E. Bagrova, A. S. Frolova, O. N. Mityaeva, P. Yu Volchkov, A. A. Deviatkin

**Affiliations:** ^1^ Institute of Translational Medicine, Pirogov Russian National Research Medical University, Moscow, Russia; ^2^ Institute of Gene Biology Russian Academy of Sciences (RAS), Moscow, Russia; ^3^ Faculty of Biology, Lomonosov Moscow State University, Moscow, Russia; ^4^ Endocrinology Research Centre, Moscow, Russia; ^5^ State Virus Collection Laboratory, Federal State Budget Institution “National Research Centre for Epidemiology and Microbiology named after Honorary Academician N F Gamaleya” of the Ministry of Health of the Russian Federation, Moscow, Russia; ^6^ Faculty of Physics, Lomonosov Moscow State University, Moscow, Russia; ^7^ Sechenov First Moscow State Medical University, Moscow, Russia; ^8^ Sirius University of Science and Technology, Sochi, Russia; ^9^ Federal Research Center for Innovator and Emerging Biomedical and Pharmaceutical Technologies, Moscow, Russia; ^10^ The Moscow Clinical Scientific Center (MCSC) named after A. S. Loginov, Moscow, Russia

**Keywords:** defective interfering particles, antiviral therapy, viruses, SARS-CoV-2, Nipah virus, poliovirus, coxsackievirus

## Abstract

Viruses are obligate parasites, that use the host’s internal metabolic systems for their own reproduction. This complicates the search for molecular targets to prevent the spread of viral infection without disrupting the vital functions of human cells. Defective interfering particles (DIPs) are natural competitors of viruses for important resources of viral reproduction. DIPs emerge during infection, originate from the normal viral replication process and inhibit its progression, making them an interesting candidate for antiviral therapy. Here we describe the biology of DIPs, advances in DIP-based antiviral technology, analyze their therapeutic potential and provide a systemic overview of existing preventive and therapeutic antiviral strategies.

## 1 Introduction

Viruses use host cellular resources for their reproduction. Infection of humans with viruses leads to the development of infectious diseases. Today, there are about 270 known viruses that have been detected in human biological material (Forni et al., 2022), that have varying clinical dynamics and possible outcomes of infection. Some of them, such as HIV (Beloukas et al., 2016), the influenza virus (Bailey et al., 2018), the measles virus (Furuse et al., 2010) and the hepatitis C virus (Razavi et al., 2020), can be transmitted from person to person and cause serious illness or death. Others, such as the Epstein-Barr virus (Damania et al., 2022; Soldan and Lieberman, 2023), adeno-associated viruses (Boutin et al., 2010) and the Torque Teno virus (Webb et al., 2020), are widespread in the human population and are not thought to pose a direct threat to the life of an infected person. In addition, cases of human infection with viruses from animals such as rabies virus (Fooks et al., 2014), tick-borne encephalitis virus (Ruzek et al., 2019) and dengue virus (Roy and Bhattacharjee, 2021) have been described, which may be fatal to the infected person but have no pandemic potential. Moreover, many viruses that circulate in the environment are still undescribed, while presenting a threat for human well-being (Anthony et al., 2013). Recent examples of these are the SARS-CoV-2 (Andersen et al., 2020) and H1N1pdm09 (Wille and Holmes, 2020) viruses, whose introduction into the human population led to the development of pandemics, resulting in the deaths of some of those infected. Relatively limited knowledge about variety of viruses and their possible health hazards creates the necessity of antiviral therapy affecting wide range of viral strains.

Currently, there is no proven universal approach that can cure or prevent multiple viral infections. However, there are several effective methods to prevent and treat some viral infections. The possibilities and limitations of such approaches are highlighted in the following sections of this review. Nonetheless, current experimental studies show that there are new therapeutic possibilities. Recently, a number of optimistic publications have appeared that suggest utilization of general principles of virus biology for development of antiviral drugs against a variety of infections on the basis of defective interfering particles (DIPs). DIPs are virus-like particles that emerge during the process of viral replication. These particles present genetic derivatives of a standard virus (STV), replicate only in its presence and consume its resources during co-infection, thereby inhibiting the replication of STV (Stauffer Thompson et al., 2009). In this way, artificially produced DIPs in high concentrations may significantly slow down the replication of original virus and be used to develop a new class of antiviral drugs. This review paper aims to summarize the experimental studies conducted on the subject of DIPs and discuss whether this method has prospects for creating perspective antivirotics.

## 2 Current prophylaxis for viral infections

The most effective existing method of preventing infectious diseases is vaccination against pathogens. According to the World Health Organization, vaccines save 3.5-5 million lives every year from diseases such as diphtheria, tetanus, whooping cough, influenza and measles (Gebre et al., 2021). The main principle of any vaccine is to train the immune system to eliminate the selected pathogen. After administration, the vaccine components are recognized by T lymphocytes. Activated T cells interact with B lymphocytes to cause their differentiation. As a result, the B cells are enabled to produce antibodies. Finally, the T and B cells acquire the ability to reactivate when encountering the pathogen in the future (Lu et al., 2018). There are different types of vaccines based on different principles: live attenuated vaccines, inactivated pathogens, subunit vaccines (Schiller and Lowy, 2015), nucleic acid vaccines (Bahl et al., 2017).

Live attenuated viruses are obtained by extended passaging - after the virus has passed through a series of cell cultures (Alvarez et al., 2020) or animal embryos (Xia et al., 2018). After these passages, the virus is weakened and can no longer replicate effectively in human cells (Gebre et al., 2021). Rotarix, the oral rotavirus vaccine, for example, was developed by passaging the rotavirus strain 89-12 isolated from a stool sample. Initially, this wild-type strain was passaged 33 times in African green monkey kidney cells. The derivative of strain 89-12 was then passaged a further 10 times in Vero cells to produce the final lyophilized vaccine (RIX4414) (Ward and Bernstein, 2009). The human immune system usually eliminates the pathogen before it leads to the development of the disease. Although these vaccines elicit strong immune response, injection of a live pathogen can pose a risk to humans with immunodeficiency conditions (Gebre et al., 2021). For example, after receiving the polio vaccine, one in 750,000 children developed paralytic poliomyelitis (Kew et al., 2005). In addition, during infection by attenuated virus, spontaneous mutations in the viral genome may enhance its virulence (Zhou et al., 2016). At the same time, an approach to prevent reversion to virulence of the live attenuated polio vaccine was recently demonstrated (Yeh et al., 2020).

In the past, the pathogenic component of inactivated vaccines was obtained by infecting primary cells, tissues, fertilized eggs or whole organisms with a pathogen. To date, the most common approach is to propagate the pathogen in cell lines (Sanders et al., 2015). The IMOVAX rabies vaccine, for example, is produced by harvesting human diploid cells, MRC-5, infected with strain PM-1503-3M and then concentrated by ultrafiltration and inactivated with beta-propiolactone (Wu et al., 2011). The accumulated mass of the pathogen may be inactivated in various ways, for example by formaldehyde, glutaraldehyde, 2,2-dithiodipyridine, β-propiolactone, binary ethyleneimine, pH, temperature, gamma irradiation, ultraviolet light (Delrue et al., 2012). As a result, all antigens are present in the inactivated vaccine and produce a broad immune response.

Subunit vaccines contain either a single antigen or a combination of multiple pathogen antigens sufficient to elicit an immune response (Moyle and Toth, 2013). The term “subunit vaccines” may refer to vector vaccines where the vector is used to deliver a fragment of the viral genome, recombinant viral proteins. Recombivax-HB, for example, consists of the surface antigen (HBsAg) of the hepatitis B virus, which is produced in yeast cells. A portion of the hepatitis B virus gene encoding HBsAg is cloned into yeast and the non-infectious subunit of the hepatitis B vaccine is produced from cultures of this recombinant yeast strain (Zhao et al., 2011).

In addition, a new class of vaccines - mRNA vaccines - has recently been introduced into clinical practice in the context of the COVID-19 pandemic. Pfizer-BioNTech’s vaccine (BNT162b2), for example, is based on the administration of the mRNA encoding the SARS-CoV-2 spike protein into the human organism (Stuart, 2021). In addition, there are genetic vaccines based on the transfection of nucleic acids into eukaryotic cells, whereupon the human cell produces a viral protein that is destroyed by the immune system (Verbeke et al., 2019).

It should be noted that other promising approaches to creating vaccines are currently being developed. For example, defective flaviviruses, which genome lacked the capsid gene elicited immune response (Mason et al., 2006). Such live defective viruses are not able to reproduce themselves in human cells, demonstrating high safety. Another actively researched method of producing new vaccines is virus-like particles (VLPs), which themselves consist of viral or artificial proteins without a nucleic acid incorporated into the VLP, and are capable of eliciting an immune response (Mohsen and Bachmann, 2022). The Cervarix vaccine against human papillomavirus types 16 and 18, for example, consists of the main capsid protein L1 virus-like particles (VLPs) formulated in an ASO4 adjuvant. It is produced using insect cells infected with recombinant baculoviruses (Monie et al., 2008).

In summary, today vaccines are being developed for a wide variety of viral infections. Nevertheless, this approach cannot be considered universal, as the effectiveness of each vaccine is highly limited to a certain group of the viruses. Also, due to mutative processes, vaccines have to be renewed to keep their efficiency when counteracting the newly emerged strains. Additionally, independently of a vaccine type, when made from a killed pathogen or a part of it, its virus does not actively replicate in the organism, so a single dose of administered vaccine may not be sufficient to build long-term protection. Therefore, booster vaccinations are often necessary.

## 3 Current therapy for viral infections

There are two alternative approaches for antiviral drug therapy: targeting the virus itself or host cell factors. Molecular targets in viruses generally include capsid elements, polymerases, proteases, nucleoside and nucleotide reverse transcriptases, and integrases.

Inhibition of viral DNA replication can be achieved by chain termination using nucleotide derivatives such as aciclovir (ACV), valaciclovir, ganciclovir, penciclovir and others (Fyfe et al., 1978; Elion, 1982; Balfour, 1983; Boyd et al., 1987; Anderson et al., 1995; Tyring, 1995; Wutzler, 1997). These compounds can be primarily phosphorylated by viral thymidine kinase, which gives them high antiviral specificity. Foscarnet (phosphonomethyl acid) is another replication inhibitor that mimics pyrophosphate and selectively inhibits the pyrophosphate binding site at concentrations that inhibit human DNA polymerases to a lesser extent (Safrin et al., 1991). Targeted inhibition of DNA has disadvantages such as the emergence of drug resistance. In particular, herpesvirus and cytomegalovirus particles can develop mutant protein kinases (thymidine kinase and UL97 protein kinase, respectively) that make them resistant to corresponding drugs (Chou, 2008). Similar approaches have also been used to inhibit RNA polymerisation. Remdesivir, for example, has a broad spectrum of activity against coronaviruses and filoviruses (Radoshitzky et al., 2023). Nevertheless, in addition to drug resistance, this drug has adverse effects such as respiratory failure and impairment of other organs (Ansems et al., 2021).

Nucleotide polymerisation is not the only target process of antiviral treatment. Amantadine and rimantadine appear to suppress replication of influenza infection by blocking the M2 ion channel protein (Alves Galvão et al., 2014). Antiretroviral therapy can involve reverse transcription inhibitors as well as protease inhibitors, integrase inhibitors, fusion and binding inhibitors (Menéndez-Arias and Delgado, 2022) (abacavir, darunavir, enfuvirtide and others). Drug resistance is a challenge for these therapies. In particular, antiretroviral drugs with low genetic barriers to resistance that have been prescribed for many years have been reported to have high levels of transferred drug resistance (Baxter et al., 2015; Bai et al., 2020). Examples include K103N/S, Y181C/I and G190A/S, which are associated with resistance to first-generation non-nucleoside reverse transcriptase inhibitors, and M184I/V, which is associated with resistance to nucleoside reverse transcriptase inhibitors (Menéndez-Arias, 2013). Adverse effects are also a major challenge. For example, tenofovir disoproxil fumarate can cause life-threatening side effects such as lactic acidosis, liver and kidney toxicity. At the same time, recently developed integrase inhibitors with lower renal and bone toxicity induce oxidative stress, fibrosis, adipogenesis, lipogenesis and insulin resistance, leading to weight gain and obesity (Gorwood et al., 2020; Scarsi et al., 2020; Tao et al., 2020). Among the integrase inhibitors, dolutegravir also has neuropsychiatric side effects (Apostolova et al., 2015).

Some challenges can be overcome by strengthening host defenses, particularly through the use of drugs that target immune responses, regulate cytokine storms and modulate epigenetic changes. Host-targeted drugs may have a broader spectrum of action than virus-targeted drugs. Interferons are also used in antiviral therapy. Interferon alpha has been shown to be effective in treating diseases caused by human herpes virus 8, papilloma virus (Kaposi’s sarcoma), and hepatitis B and C viruses. Interferons work by modulating the host’s immune response to infection. They stimulate both macrophages and NK cells to trigger an antiviral response involving the IRF3/IRF7 antiviral pathways (Zhou et al., 2015). However, modulation of the immune response by interferons also has its drawbacks. Many viruses have evolved to switch off interferon-mediated signaling pathways and interferon-induced antiviral proteins, for example, by blocking apoptosis and thus preventing interferon-triggered containment of infection. In particular, numerous viruses have been shown to inhibit STAT1/2, PKR and interferon-stimulated genes (ISGs) (Davies et al., 1993; Gale and Katze, 1998; Chatterjee-Kishore et al., 2000; Howe et al., 2000; Stojdl et al., 2000; Seet et al., 2003; Shaw et al., 2004; Stuart et al., 2016; García-Sastre, 2017). In addition, the arsenal of antiviral drugs includes monoclonal antibodies (mABs). The mechanism of action of mABs is different. On the one hand, mAbs specifically target and bind to viral particles, preventing them from entering the target cells and causing infection (Pantaleo et al., 2022). On the other hand, mABs modulate the response of the human immune system to infection (Pantaleo et al., 2022), which is a challenging task and can lead to complications. It should be noted that in infections such as SARS-CoV-2, the patients with the most severe lung involvement are not directly damaged by the infection, but by the hyperactivation of the immune system, which primarily contributes to the tissue damage (Tay et al., 2020).

Despite significant breakthroughs in antiviral therapy, it still faces many obstacles. The development of viral therapeutics is time-consuming and requires targeted investments. Antiviral resistance is the major problem which is caused by common mutations that alter the molecular targets of antiviral drugs. Even potentially more universal host-specific drugs can cause resistance under certain conditions, specifically in the case oflong-term selection pressure that allows the virus to mutate and adapt to treatment. Viruses have evolved to counteract immune responses that can render established therapies ineffective.

## 4 Biology of defective interfering particles

Defective particles, containing viral genome with point mutations or deletions that disrupt the viral reproduction cycle, occur in every reproductive cycle of all viruses, especially viruses containing RNA. It should be noted that there are several mechanisms for the formation of such particles. In some cases, defective particles compete with wild-type viruses for cell resources and hinder their replication (Xiao et al., 2021). In other cases, defective particles have no effect on further viral infection development (Tuchynskaya et al., 2021). Moreover, sometimes even in the case of the large deletions in the genome, such as complete deletions of the Internal Ribosome Entry Site (IRES) some viruses can maintain their replication process (Murray et al., 2004). In other words, defective viral particles do not always interfere with the reproduction of the wild-type virus.

**Figure 1:**
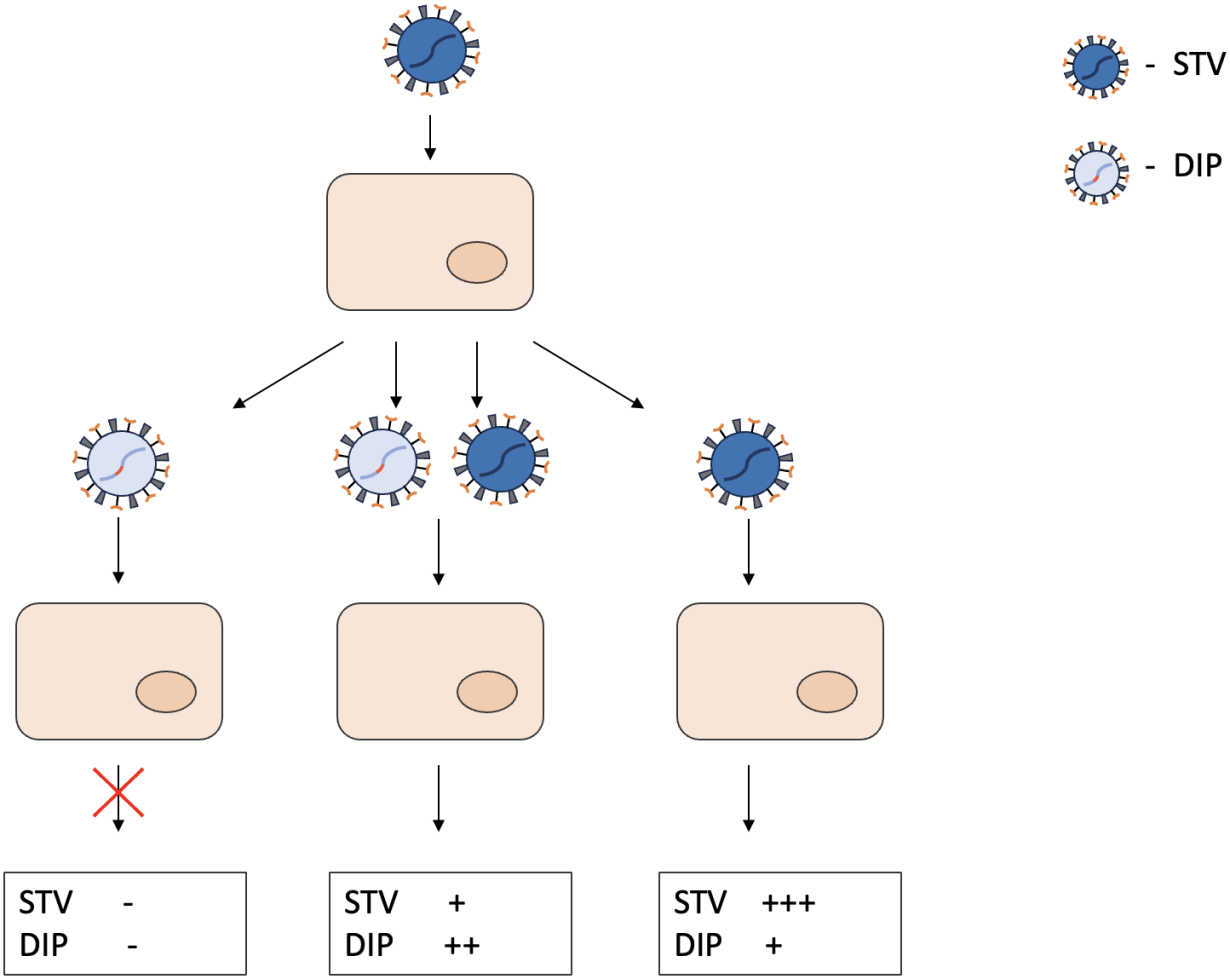
General principles of DIPs biology and resulting DIP/STV ratios in the cell culture. DIPs have a truncated genome and the gene responsible for replication is damaged. When infected with DIPs only, the cell does not generate any more viral particles. In case of coinfection with DIPs and STV, both viral particles proliferate in the cell, competing for resources in the process. When infected with STV only, mistakes in the replication process lead to formation of different DIPs. “-” indicates the absence of STV or DIPs production. “+” indicates the intensity of STV or DIPs production.

DIPs or DI particles are a type of defective particles that structurally resemble STVs but contain only a portion of the viral genome (Huang and Baltimore, 1970). DI particles are produced when cells are infected with STVs (**Figure 1**). Replication of wild-type viruses results in a genetically diverse group of defective genomes with mutations in essential genes. The formation of DI particles is based on a specific set of defective genomes that are capable of replication and subsequent assembly. Due to the mutations in the genome, the successful propagation of DIPs requires the presence of STVs. The inevitable competition for resources between STVs and DIPs within the cells leads to excessive production of DI particles. The more DIPs are produced, the fewer STVs are present. Since the replication of DIPs depends on STVs, the number of DI particles also decreases until only a few DI virions and STVs remain. Compared to other subviral pathogens, DIPs have the following characteristics: they contain a portion of a standard genome, replicate only in the presence of wild-type viruses, are composed of structural proteins from STVs and interfere with the production of homologous STVs. The sum of the above characteristics defines the complex term DIPs.

It is now recognized that most viruses are capable of producing DIPs (Marriott and Dimmock, 2010). DIPs have been demonstrated for both DNA and RNA, double-stranded and single-stranded viruses infecting a wide variety of hosts (Vignuzzi and López, 2019). Recently, the ability to produce DIPs has been demonstrated for a group of viruses, which causes severe disease in humans: Nipah virus (Welch et al., 2020), Zika virus (Rezelj et al., 2021), SARS-CoV-2 (Gribble et al., 2021). The interference nature of DIPs and their wide distribution among different phyletic groups, make them a potential medication for antiviral therapy.

### 4.1 Mechanism of DVG’s generation

The key event in the occurrence of DIPs is the generation of defective genomes called defective viral genomes (DVGs). The mechanism by which DVGs are generated is different for DNA and RNA viruses, although both types use parental viral genomes as templates.

In RNA viruses, low-fidelity viral RNA-dependent RNA polymerase (RdRp) plays an essential role in the generation of DVGs. Errors introduced during replication lead to mutated (mutation rate — 10^-4^ (de Farias et al., 2017)), truncated and/or rearranged genomic sequences. During replication, RdRp can leave the donor template and resume synthesis on the acceptor strand, resulting in a defective genome (Simon-Loriere and Holmes, 2011; Poirier et al., 2016). Deleted genomes occur when the RdRp resumes the same strand at a different location in the genome. As a result, genomes with deletions (or other defects: insertions, inversions or duplications) lack genes important for self-replication, but retain terminal regions necessary for packaging and replication. Another type of recombinant DVGs are genomes with reverse complementary regions. Defective “panhandle” genomes, common in negative RNA viruses, are the result of RdRp reattaching to the nascent strand and copying the genome end. In addition, defective genomes may have longer complementary regions and resemble hairpins. Therefore, RdRp can occasionally unbind the template and induce a series of DVGs. Mutations in RdRp may increase the probability of these events (Fodor et al., 2003). Interestingly, the possible artificial induction of template switching by drugs and their use in therapy is still under investigation (Janissen et al., 2021). Although RdRp has been proposed as the main driver of DVGs, other mechanisms involving viral (Yoshida et al., 2018) and host factors (Kuniholm et al., 2022) have also been explored.

Compared to RNA viruses, DIPs of DNA viruses have not been studied as thoroughly. In addition, DNA viruses take a different approach to generating DVGs, relying more on cellular recombination (Yalamanchili et al., 1990) or repair mechanisms (Zhang et al., 2022). For example, non-homologous end joining and hypermutation by APOBEC3B, a DNA cytosine deaminase, have been suggested as a possible cause of DVGs in adeno-associated viruses (AAV) (Zhang et al., 2022) and BK polyomavirus (Addetia et al., 2021).

## 5 Mechanisms of DIPs therapeutic effect

**Figure 2:**
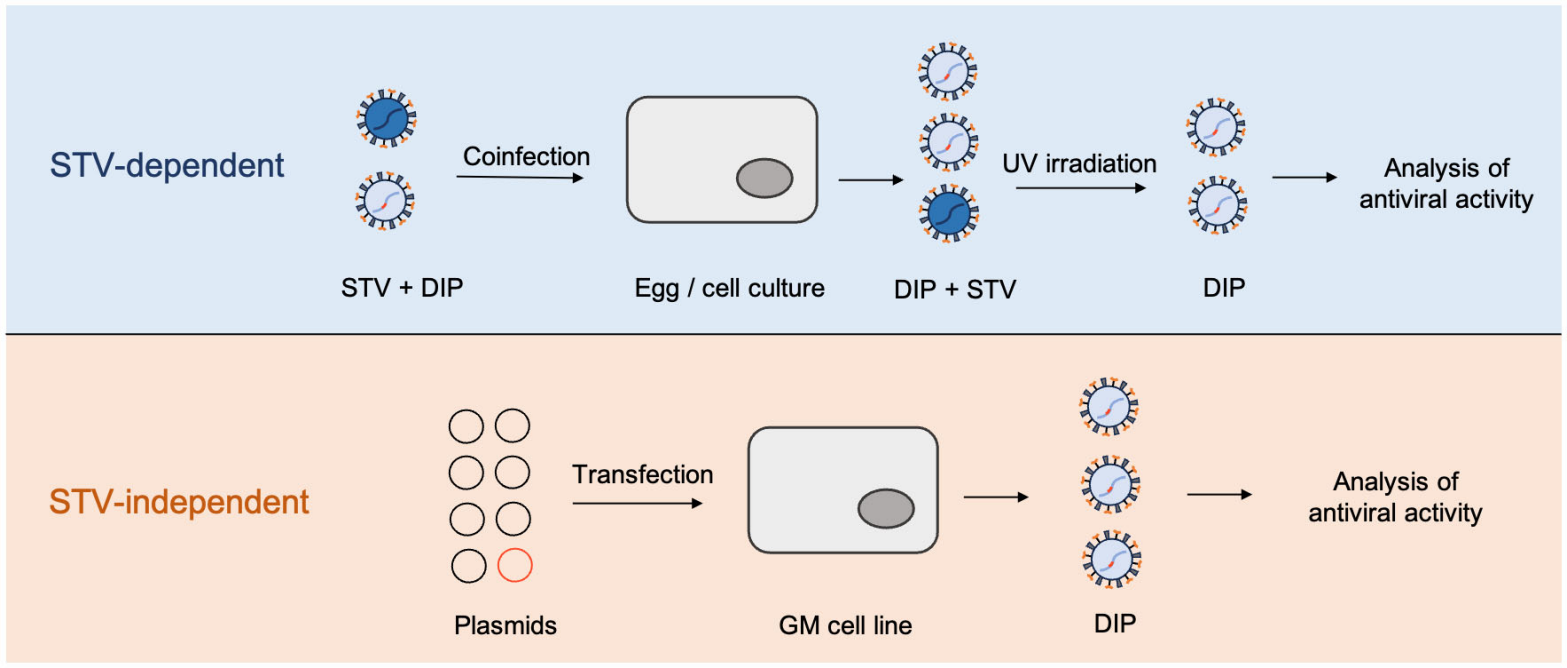
High-yield approaches to DIPs production. STV-dependent approaches require the presence of active original virus and therefore cannot be considered biologically safe. STV-independent approach illustrates the method proposed by Najat Bdeir et al. (2019). In this approach, DI-244 particles of IAV were generated solely from the plasmids, using 293T and MDCK cell lines, stably expressing codon optimized PB2; GM = genetically modified.

DIPs can significantly slow the spread of viral infections between cells (Baltes et al., 2017) because the resources of the replication or protein synthesis apparatus of viruses are used by defective particles that are not capable of self-replication or self-assembly (**Figure 2**). Suppression of replication of STVs is one of the characteristic features of DIPs. Interference phenomena mediated by DIPs can be caused by a number of reasons. In general, truncated/shortened DVGs successfully compete with complete wild-type genomes for viral replication and structural proteins (Shirogane et al., 2021). Eventually, the decline in standard viral genomes reduces the productive infection. In addition, DVGs can encode new variants of viral proteins that can reduce natural viral replication. Examples of this are the fused Nsp1-10 protein of SARS-CoV-2 (Girgis et al., 2022) or the Hyb proteins of EHV-1 (Ebner and O’Callaghan, 2006).

Two main factors determine the outcome of the infection. First, if the initial DIPs amount is significantly greater than the amount of wild-type virus, such an infection is likely to clear on its own. The extent of replication of DIPs compared to the wild-type virus during coinfection is another important factor. Even if the presence of DIPs does not eliminate the infection, it will at least impede it and give the immune system valuable time to develop a specific response.

In addition, a non-specific mechanism of action of DIPs has been proposed (Xiao et al., 2021). DVG can produce double-stranded RNA that can be recruited by the cytosolic sensors MDA-5 or RIG -I and cause the expression of interferon-stimulated genes (Hur, 2019). DVG can thus indirectly cause inflammation that inhibits replication of the wild-type virus. Thus, *in vitro* and *in vivo* studies indicate direct competition between DIPs and STV and additional indirect exclusion of the latter. This phenomenon may have potential for use in the development of antiviral drugs.

## 6 DIPs production *in vitro*

In the late 1970s, DIPs were generated as a natural byproduct of virus replication in series of infected cell passages, which was a time- and resource-consuming process that could not always guarantee the success of DIPs generation (Guild and Stollar, 1975; Stark and Kennedy, 1978). Currently, for a small-scale production synthetic DIPs are constructed *in vitro* via a reverse genetics approach - from several plasmids, encoding portions of the wild-type virus genome (Neumann et al., 1999; Hoffmann et al., 2000). Site-directed mutagenesis and inverse PCR are usually used for inactivation of the gene responsible for replication. After generation, plasmids are transfected into the cell line together with a plasmid responsible for the synthesis of a missing protein (Yamagata et al., 2019). An alternative approach involves the usage of lentiviral and retroviral vectors delivering DIPs RNA (Lin et al., 2022). Further, the DI virus is propagated in the cell culture, titrated by plaque assay, and can be used for further experiments. To obtain new, not yet discovered DIPs, a different approach involving the infectious STV is used. The DI virus can be yielded from a STV-infected single-cell virus isolate and then enriched by the cell culture. In this way, Kupke et al. discovered a novel unconventional influenza A virus derived DIPs, named OP7 virus, which had numerous point mutations instead of deletions in the genome and demonstrated efficient inhibition of virus release during STV infection (Kupke et al., 2018).

**Figure 3:**
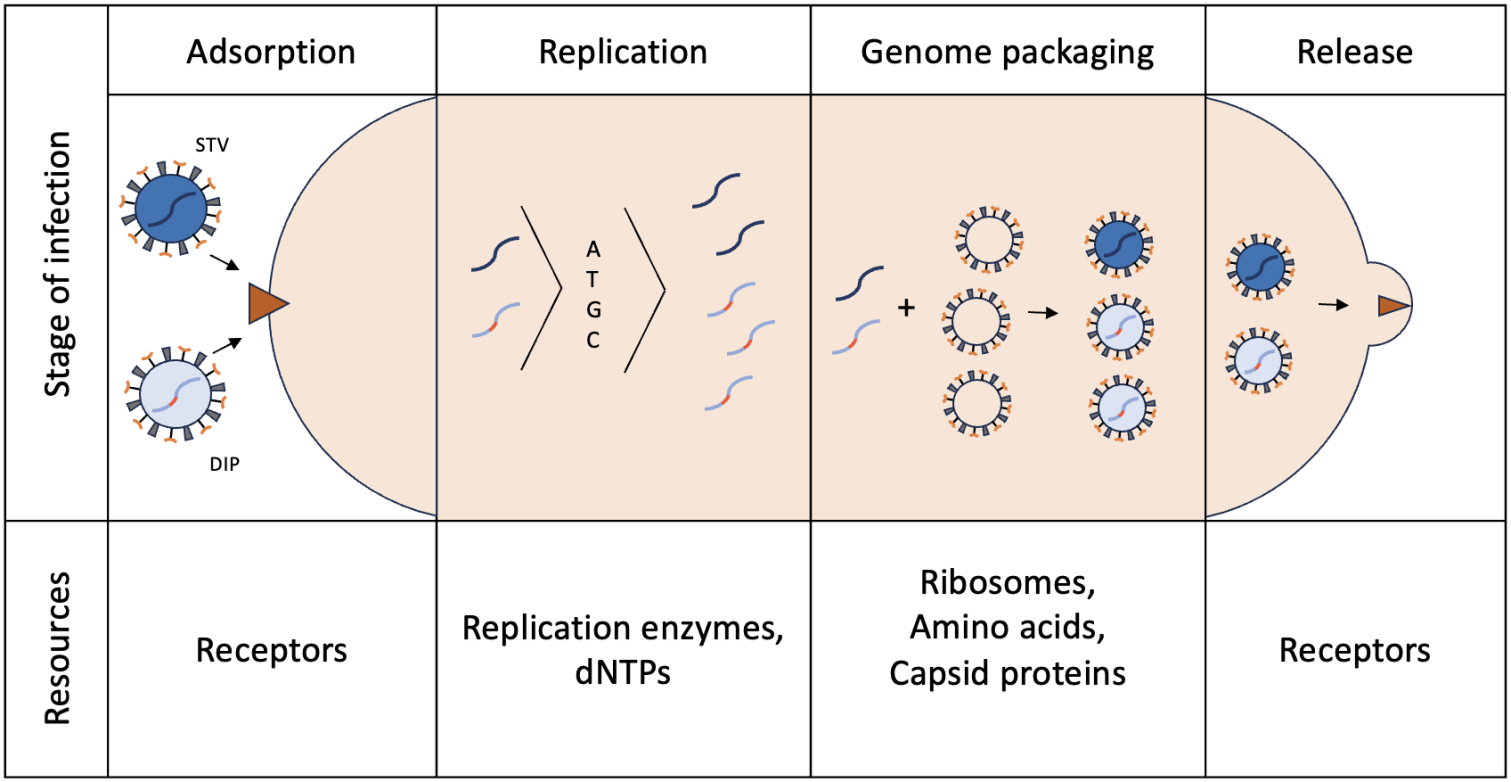
Stages of viral infection and competition resources between STV and DIP. During coinfection, STV and DIPs compete for distinct cellular/viral resources at every stage of the infection. In this way DIPs limit reproduction and expansion of STV.

Large-scale manufacturing of DIPs would require continuous production, sufficient product yield, and batch-to-batch consistency of quality. Current models for the cultivation of high-yield DIPs are generally limited to cell culture-based cultivation of the STV and DIPs in bioreactors followed by particle identification by segment-specific reverse transcription-PCR (RT-PCR) (**Figure 3**). The product is then UV-irradiated to eliminate the possible contamination with the infectious virus. Then steric exclusion or other types of chromatography can be used to purify and concentrate the UV-treated material (Hein et al., 2021c; Lin et al., 2022). The effectiveness of the resulting product is tested *in vitro* by the interference assay and *in vivo* to assess the toxicity.

Continuous cell culture systems, which allow to achieve high cell densities, are being actively developed for production of DIPs. Such systems allow continuous influx of fresh medium to the cells while the previous medium is removed and the volume of the system remains constant (Gutiérrez-Granados et al., 2018). The research group of Udo Reichl developed the models of semi-continuous and continuous influenza A virus (IAV) and DIPs production, which are based on separate cultivation of cells with their continuous “feeding” to a virus bioreactor (Tapia et al., 2019; Pelz et al., 2021). Tapia et al. developed a system for continuous DIPs production, consisting of one bioreactor, where cells are cultivated, and then being fed to two virus bioreactors, functioning in parallel. Such a system provides a single source of cells to two experimental tanks and helps to compare cultivation parameters, virus seeds and cell lines or study oscillations in virus and cell concentrations in time. The experimental model was supplemented with a mathematical model that efficiently predicted oscillations in cell population dynamics and DIPs to STV ratios (Tapia et al., 2019).

Nevertheless, considering that DIPs cultivation still requires the presence of a complementary virus to provide the missing function of replication – cell coinfection with STV and DIPs cannot be considered biologically safe and may cause difficulties in data analysis due to residual contamination with STV. Strategies for obtaining purely clonal DIPs that do not require the cultivation of STV, are currently being developed. These are based on infection and cultivation of genetically modified cell lines expressing the gene for the missing viral protein (Bdeir et al., 2019; Hein et al., 2021a). Yamagata et al. used a cell line, which stably expressed PB2 protein, to overcome the need for the use of STV during DI influenza virus generation (Yamagata et al., 2019). Recently, the proposed model has been refined to an automated perfusion process for the production of DIPs using an alternating tangential flow filtration (ATF) system for cell retention (Hein et al., 2021b). Thus, STV-independent cell culture-based DIPs manufacturing currently seems to be the most promising direction of technological development as it increases the product yield, speeds up the process, and makes it more biologically safe.

## 7 Experimental evidence of DIPs therapeutic potential

At the moment, DIPs were tested both *in vitro* and *in vivo* for a variety of viruses and demonstrated effectiveness of application in multiple studies. In *in vitro* cell culture experiments, DIPs has been shown to be effective against many viruses, such as influenza A (IAV) (Tapia et al., 2019), dengue virus (DENV), Zika virus (ZIKV), yellow fever (YFV), respiratory syncytial virus (RSV), severe acute respiratory syndrome coronavirus 2 (SARS-CoV-2) (Lin et al., 2022), mumps virus (Šantak et al., 2015), hepatitis B virus (HBV) (Yuan et al., 1998), Nipah virus (NiV) (Welch et al., 2020) and others. In addition, an ability of conditionally replicating HIV to interfere with wild-type HIV replication and spread has been demonstrated (Dropulić et al., 1996). In all cases, there is a decrease in viral replication and a decrease in viral infection. According to experiments on cell lines, DIPs from one origin may demonstrate applicability to several strains. When produced from the H1N1 serotype of IAV, DIPs effectively prevented the spread of both H1N1 and H3N2 strains (Kupke et al., 2018). Thus, it is hoped that DIPs can be used against viruses that use similar replication machinery.

The mechanisms of action of DIPs *in vivo* may vary depending on the structure of the DIPs and the route of administration. The DIPs remain in the cells they have entered after administration. If the wild-type virus infects the same cell, the DIPs may interfere with its replication. It should be noted that the diversity of DIPs is comparable to the diversity of viruses, since DIPs are descendants of the corresponding STVs that have been created by inserting fragmentary genome variants into the viral capsid instead of the complete genome. This requires that the DIPs nucleic acids were recognised and replicated by viral polymerases and that the DIPs nucleic acids were packaged into the viral capsid. Despite the enormous diversity of DIPs in terms of virion structure, their mechanism of action is therefore based on competition with viral wild-type nucleic acids during replication and assembly of the virion. This interaction is based on conservative signaling for recognition by polymerases and assembly of the virion. For this reason, DIPs inhibit not only STVs, but also similar viruses of the same species that contain point mutations, as well as viruses of a different species. Recent studies showed a remarkable antiviral effect of DIPs against other types of viruses that were heterologous to DIP-related STV. In this way, IAV-derived DIPs exert *in vitro* and *in vivo* antiviral effects against a broad spectrum of viruses, such as influenza B, SARS-CoV2, yellow fever, Zika virus, pneumovirus infections and others (Scott et al., 2011a; Xiao et al., 2021; Pelz et al., 2023).

In recent years, the exact molecular mechanisms of the antiviral effect of DIPs have gradually been deciphered. Scott et al. not only demonstrated the efficacy of IAV-derived 244/PR8 against influenza B *in vivo*, but also emphasized the importance of type I IFN in regulating this process (Scott et al., 2011a). Indeed, *in vitro* experiments have shown that in cells with deficient IFN production, the antiviral activity of IAV-derived DIPs is not observed (Pelz et al., 2023). Pelz et al. have also demonstrated the involvement of the JAK/STAT signaling pathway in the IFN-mediated development of antiviral activity of cells against ZIKV (Pelz et al., 2023). In this way, the mechanisms of antiviral protection by DIPs vary from direct competition with genomes of homologous viruses to the broad effect of IFN-mediated immunity activation against heterologous viruses.

Intranasal administration of DIPs is widely used and is suited for the treatment of respiratory diseases caused by viral pathogens. In the *in vivo* experiments, mice were infected with influenza A virus and then DIPs were administered intranasally. The presence of DIPs attenuates the course of the disease, induces an immune response and increases the survival rate of DIP-treated animals (Scott et al., 2011b; Hein et al., 2021a). The efficacy of DIPs is demonstrated for *in vivo* models of many other viral respiratory infections (**Table 1**). For example, intranasal administration of therapeutic DIP-containing lipid nanoparticles (SARS-CoV-2 DIPs) to Syrian golden hamsters has been shown to reduce viral load, decrease proinflammatory cytokines and prevent lung injury). Viral infections of the respiratory tract are ubiquitous and often seasonal. The variability and adaptation of viruses poses a challenge for vaccine development. The defective interfering particles show good results in treating respiratory viral infections in animal models, opening up the potential for their use in the clinic. Another DIP, produced from the poliovirus genome by deleting the capsid coding region, effectively protected mice from SARS-CoV infection. It was shown that although this type of DIPs was capable of replication, this process occurred exclusively in the initially infected cells at the administration sites (Xiao et al., 2021). Although the infection was localized, it led to a broad activation of innate and adaptive immunity, resulting in successful antiviral protection.

**Table 1:** Therapeutic effects of DIPs *in vivo* ^*^.

ST Virus	DIPs composition	*In vivo* model	The route of DIPs administration	Results	Reference
SARS-CoV-2	SARS-CoV-2-based TIPs (TIP1 and TIP2) encoded some parts of 5′- UTR, ORF1ab, the 3′-UTR. TIP2 was larger than TIP1 and contained a part of N-protein in addition.	Syrian golden hamsters	Intranasal	Administration of TIPs reduced viral load in the lungs and suppressed inflammation and severe disease when given before or after infection. In addition, TIPs reduced the transmission of SARS-CoV-2.	(Chaturvedi et al., 2021, 2022)
NiV	NiV TIP with multiple DIPs: DI-07, DI-14, DI-35 were variants of copyback of different length and DI-10 with deletion.	Aura Syrian hamsters	Intraperitoneally	Administration of TIPs improved clinical outcomes and reduced NiV-associated mortality. Overall, 80% of hamsters survived on DI-07 and DI-14 treatment (60% and 40% of them were asymptomatic, respectively) and 70% on DI-35 and DI-10 (40% of each group was asymptomatic). Without such treatment, 90% of the hamsters became fatally ill with NiV.	(Welch et al., 2022)
			Intranasal	Protection was provided. However, the dose of TIPs was lower than intraperitoneal administration. Hamster survival was also lower with intranasal treatment. 80% of hamsters survived treatment with DI-70, 75% with DI-14 and DI-35 and 62.5% with DI-10 (20%, 62.5% and 12.5 were asymptomatic, respectively). At the same time, only 75% of the hamsters developed fatal NiV disease.	
PV1, CVB3, IAV, SARS-CoV-2	PV1 eTIP1 with replacing P1 region	Mice	Intraperitonial	Coinoculation of PV1 with eTIP1 significantly attenuated the disease and protected 80–90% of mice from lethal infection. CVB3 therapeutic postexposure eTIP1 (CVB3 infection and then, after 1 or 2 days of eTIP administration) protection was shown.	(Xiao et al., 2021)
			Intranasal	Co-inoculation of eTIP with PV1 protected against death. Prophylactic pre-exposure to PV1 (administration of eTIP1 and then, after 2 days, PV1 infection) and therapeutic postexposure (PV1 infection and then, after 1 and 2 days, administration of eTIP1) to eTIP1 also protected 90% of mice or caused significant protection. Following IAV exposure, eTIP1 protection (PR8 infection and then, after 1 day, eTIP1 administration) was demonstrated. Pretreatment with eTIP1 (eTIP1 administration and then, after 1 day, SARS-CoV-2 infection) reduced SARS-CoV-2 titres and viral RNA copies and reduced immunoreactivity in lung and brain. SARS-CoV-2 eTIP1 before exposure (SARS-CoV-2 infection and then, after 1 day or 1 and 2 days) also provided protection.	
IAV	IAV DIPs with deletion within segment 1 (DI244)	D2(B6).A2G-*Mx1^r/r^* mice	Intranasal	No toxic effects were observed in mice treated with DIP. In mice infected with a lethal dose of PR8 IAV, less body weight loss was observed when treated with DIP. All animals survived.	(Hein et al., 2021a)
IAV	IAV DIPs ОР7 with 37 point mutations in segment 7	D2-*Mx1^r/r^* mice	Intranasal	No toxic effects were observed. All mice infected with a lethal dose of IAV survived the infection after OP7 co-treatment.	(Hein et al., 2021c)
IAV	IAV segment 1 244 DI RNA in a cloned 244/PR8 virus	Ferrets	Intranasal	The efficacy of DI 244 compared to oseltamivir has been proven. Both DIPs and oseltamivir reduced infectivity and allowed adaptive immunity to develop. But only DIPs treatment significantly reduced weight loss, promoted better weight gain, reduced respiratory disease and reduced infectivity.	(Dimmock et al., 2012)
SFV	SFV DIPs: p13a, p4, p5. The number means the sum of undiluted passages. The letter indicates stocks.	CFLP mice	Intranasal	p13a protected the majority of mice (60%). The mice became immune to subsequent SFV infection. p4 protected the mice to a similar extent as p13a, but the mice remained susceptible to infection. p5 did not protect.	(Barrett and Dimmock, 1984)

^*^ SARS-CoV-2, Severe acute respiratory syndrome-related coronavirus 2; NiV, Nipah virus; PV1, Poliovirus type 1; CVB3, Coxsackievirus B3; SFV, Semliki Forest virus.

Another group of diseases for which the efficacy of DIPs has been demonstrated in animal models are neurological viral infections. In a Syrian hamster model of lethal Nipah virus, intraperitoneal or intranasal administration of DIPs particles reduces disease severity and overall lethality (Welch et al., 2022). The use of DIPs of Semliki Forest virus in infected mice causes a marked decrease in virus replication in the host and prevents lethal encephalitis (Barrett et al., 1981). Central nervous system infection caused by intranasal administration of vascular stomatitis virus in mice (Chaturvedi et al., 2021) can be effectively eliminated by intranasal administration of DIPs (Cave et al., 1984).

The dynamics of DIPs and STV coinfections are complex and have been extensively studied using both experimental and mathematical approaches. Recent mathematical modelling efforts have provided valuable insights into the competitive interactions between DIPs and STVs and the conditions under which DIPs can effectively inhibit viral replication. For example, a modelling study (Fatehi et al., 2021) has shown that therapeutic interfering RNAs containing multiple dispersed RNA packaging signals and a replication signal for the viral polymerase, but lacking any protein-coding information, significantly suppress STV. The other theoretical study (Karki et al., 2022) compared the dynamics of DIPs and STVs in the presence of adaptive and innate immunity. According to these results, DIPs significantly suppressed viral load. Although DIPs slowed down the immune response, the combined effect of DIPs and immunity was still beneficial. In addition, Liao et al. (2016) used a mathematical model to show that counting DIPs based on the reduction of STV yield (Bellett and Cooper, 1959) is suitable for counting influenza A DIPs. These models can provide an important framework for understanding how DIPs can be used in therapeutic contexts. The integration of such mathematical perspectives complements the experimental results and provides a holistic understanding of DIPs mechanisms and their potential for antiviral therapies.

## 8 Conclusions

**Figure 4:**
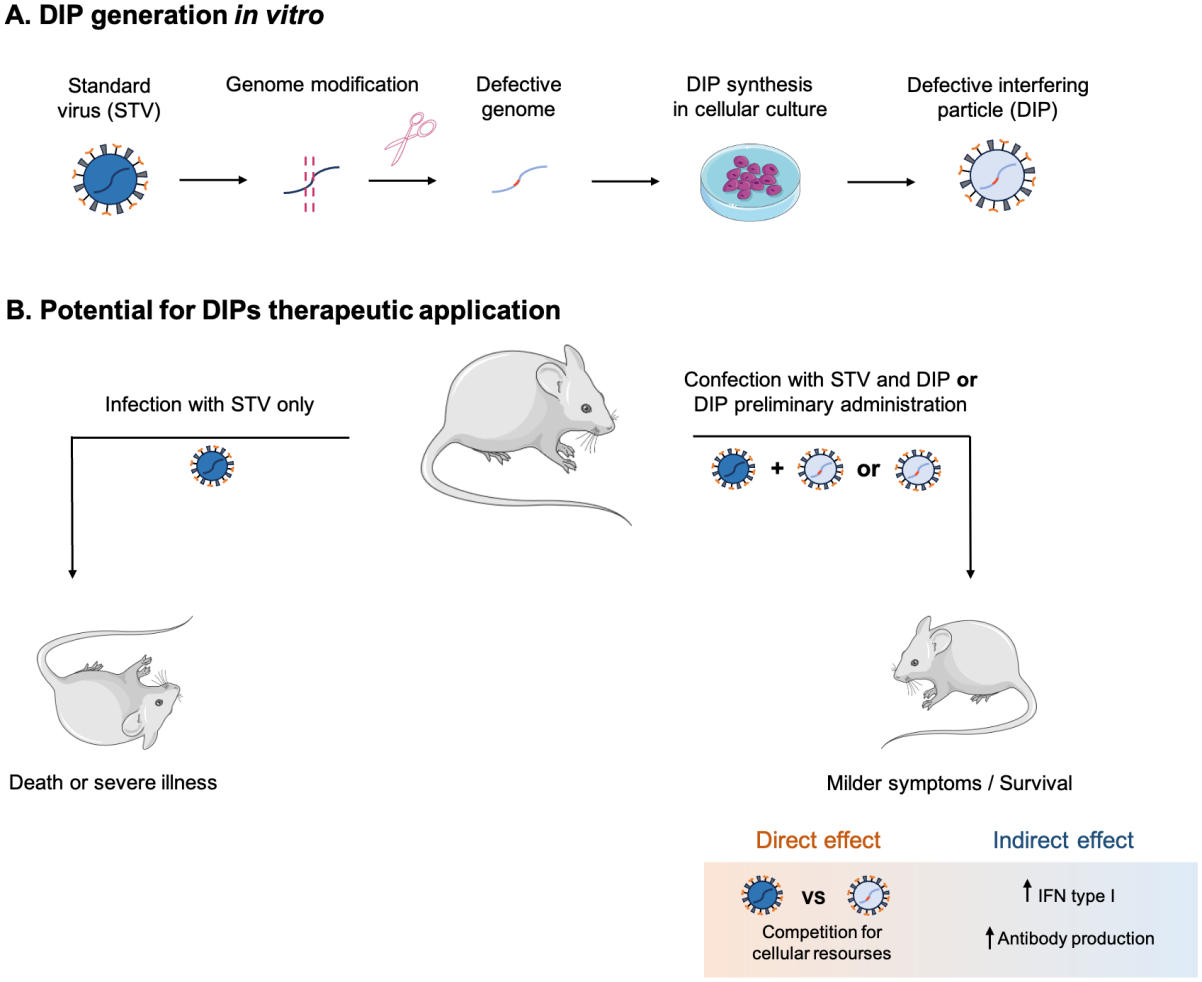
Mechanisms of DIPs production *in vitro* **(A)** and their therapeutic effect **(B)**.

DIPs derived from specific viruses are promising as antiviral agents because they compete with STVs for host cell resources, stimulate immune responses and potentially target multiple STV variants (**Figure 4**). This universality could significantly expand the armory against infections such as influenza, for which there is no universal vaccine or therapy. Advances in reverse genetics offer prospects for broader application of DIP, although its ability to inhibit a broad spectrum of viruses is not yet proven. However, there are other challenges. Prolonged use could lead to viral recombination, DIP-mediated degradation of antibodies and impeded clearance of infection, raising concerns about chronic pathology. In addition, DIPs are unsuitable for prophylaxis and ineffective in late-diagnosed infections such as Nipah. Overcoming these challenges will be critical to realizing the full potential of DIPs.DIPs DIPs DIPs.
